# Structural Probing and Molecular Modeling of the A_3_ Adenosine Receptor: A Focus on Agonist Binding

**DOI:** 10.3390/molecules22030449

**Published:** 2017-03-11

**Authors:** Antonella Ciancetta, Kenneth A. Jacobson

**Affiliations:** Molecular Recognition Section (MRS), Laboratory of Bioorganic Chemistry, National Institutes of Diabetes and Digestive and Kidney Diseases, National Institutes of Health, Bethesda, MA 20892, USA; antonella.ciancetta@nih.gov

**Keywords:** G protein-coupled receptor, site-directed mutagenesis, homology modeling, adenosine receptor, structure-activity relationship, nucleoside, docking, molecular dynamics, agonist, drug discovery, virtual screening

## Abstract

Adenosine is an endogenous modulator exerting its functions through the activation of four adenosine receptor (AR) subtypes, termed A_1_, A_2A_, A_2B_ and A_3_, which belong to the G protein-coupled receptor (GPCR) superfamily. The human A_3_AR (hA_3_AR) subtype is implicated in several cytoprotective functions. Therefore, hA_3_AR modulators, and in particular agonists, are sought for their potential application as anti-inflammatory, anticancer, and cardioprotective agents. Structure-based molecular modeling techniques have been applied over the years to rationalize the structure–activity relationships (SARs) of newly emerged A_3_AR ligands, guide the subsequent lead optimization, and interpret site-directed mutagenesis (SDM) data from a molecular perspective. In this review, we showcase selected modeling-based and guided strategies that were applied to elucidate the binding of agonists to the A_3_AR and discuss the challenges associated with an accurate prediction of the receptor extracellular vestibule through homology modeling from the available X-ray templates.

## 1. Introduction

**Figure 1:**
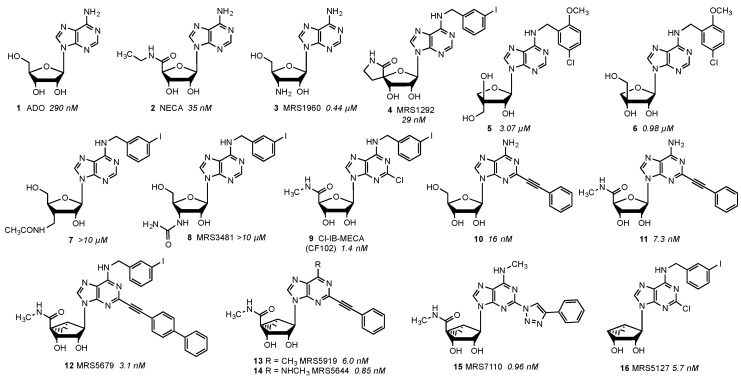
Selected representative adenosine receptor subtype A_3_ (A_3_AR) nucleoside ligands, including ribose, modified ribose and (*N*)-methanocarba derivatives (containing a bicyclic substitution of ribose in **12**–**16** that locks the conformation). Ki values in the wild-type human A_3_AR (hA_3_AR) are shown [14,18,21,22,23,24,25,26,27,28,29]. Most are agonists, but **4**–**6** and **16** display less than full efficacy.

The nucleoside adenosine (**1**, Figure 1) is an endogenous modulator regulating numerous physiological functions through the activation of four adenosine receptor (AR) subtypes, termed A_1_, A_2A_, A_2B_ and A_3_, that belong to the G protein-coupled receptor (GPCR) superfamily. Generally, ARs are involved in cytoprotective functions, although each AR subtype is characterized by distinct tissue localization and biochemical pathway activation that give rise to a unique pharmacological profile [1].

**Figure 2:**
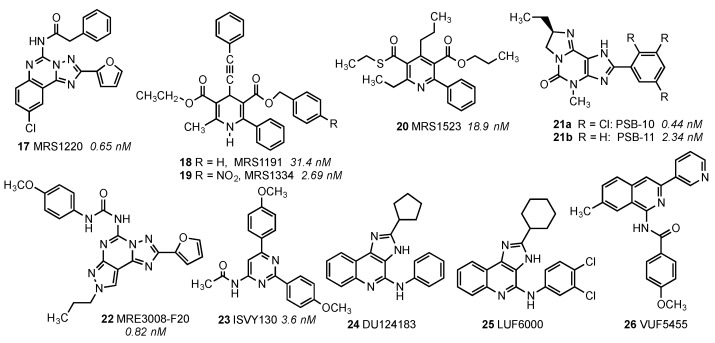
Selected representative nonnucleoside A_3_AR antagonists **17**–**23** and allosteric enhancers **24**–**26**. Antagonist Ki values in the hA_3_AR are shown [1,6,29,30,31].

The human A_3_AR (hA_3_AR) is expressed at the highest levels in liver, lung and immune cells [2], but lower levels are also detected in other tissues, including the heart and brain [3]. Small molecule modulators targeting the A_3_AR have therefore been sought for their potential application as anti-inflammatory, anticancer, and cardioprotective agents. In particular, A_3_AR agonists based on the adenosine scaffold emerged as promising antinociceptive agents in different preclinical models of chronic pain [4] and in clinical trials as agents for treating rheumatoid arthritis, psoriasis and hepatocellular carcinoma [5]. A_3_AR antagonists based on several heterocyclic scaffolds were suggested as potential candidates for the treatment of glaucoma [6,7] and respiratory tract inflammation such as asthma [8]. The structures of selected agonists, antagonists, and positive allosteric modulators (PAMs) of the A_3_AR that will be discussed below are shown in Figure 1 and Figure 2.

From the structural point of view, the A_3_AR is a cell membrane receptor consisting of 318 amino-acid residues arranged in the typical GPCR architecture characterized by seven transmembrane α-helices (TMs) connected by three intracellular loops and three extracellular loops (ELs). The N-terminal and C-terminal domains face the extracellular and intracellular sides, respectively, and the C-terminal domain of the A_3_AR, to a greater extent than other ARs, presents multiple serine and threonine residues representing potential phosphorylation sites, which may play a role in receptor desensitization upon activation by an agonist [9].

Over the years, structure-based molecular modeling techniques, and in particular molecular docking, have been increasingly applied to: (1) rationalize the structure–activity relationships (SARs) of newly introduced ligands at the A_3_AR; (2) guide the subsequent lead optimization; and (3) decipher the role of specific residues in ligand binding and receptor activation through the interpretation of site-directed mutagenesis (SDM) experiments [10,11,12,13,14,15,16,17]. Recently, molecular dynamics (MD) analysis has been applied to elucidate molecular recognition at the A_3_AR [18,19,20]. As the three-dimensional A_3_AR structure has not yet been solved, the accuracy of the predictions made with these methodologies, especially in the decades preceding the first disclosure of the structure of the hA_2A_AR [10], a close homologue, has strongly relied upon the quality in terms of sequence identity and resolution of the available templates [11,12].

The availability of three-dimensional hA_2A_AR structures in complexes with both agonists and antagonists contributed to a greater understanding of ligand recognition at ARs, thus leading to increased application of structure-based drug design (SBDD) techniques to ARs. Nonetheless, as we will discuss in the following sections, accurate hA_3_AR modeling is still challenging due to the inherent difficulties in correctly envisaging the impact on ligand binding of the highly plastic extracellular vestibule and identifying hits selective for the intended subtype during virtual screening (VS) campaigns. In this review, we showcase selected modeling and modeling-guided strategies that have been applied over the years to address the abovementioned issues, with a particular emphasis on elucidating agonist binding, as the progress of molecular modeling techniques in describing antagonist binding has been recently and extensively reviewed elsewhere [13].

Most hA_3_AR antagonists (**17**–**23**, Figure 2) were discovered empirically, by one of three methods: (1) structural modification of a known AR antagonist to bind selectively to the hA_3_AR (e.g., **17** and **22** from [1,2,4]triazolo[1,5-*c*]quinazolin-5-amine and **21a** and **21b** from 8-phenylxanthines); (2) VS of medium to large chemical libraries to identify and optimize binding hits (1,4-dihydropyridines **18** and **19**, pyridine **20** and pyrimidine **23**); and (3) modification of agonist ligands to reduce relative efficacy in receptor activation without losing binding affinity or A_3_AR selectivity (e.g., antagonist **4**) [6,21,28,29,30]. Typically, radiolabeled ^125^I agonist I-AB-MECA is used in binding assays [14], but antagonist [^3^H]**22** has also been utilized. The ^125^I nucleosides I-AB-MECA and MRS5127 **16**, as a 4′-truncated derivative [28], were found to bind to A_3_AR species homologues equipotently. However, a wide variation of affinities between species was noted for nonnucleoside A_3_AR ligands [6], including the prototypical AR antagonists, alkylxanthines, with affinity at hA_3_AR usually much greater than at rat A_3_AR. Among the antagonists shown in Figure 2, only the pyridine derivative MRS1523 **20** is reported to bind with sub-µM affinity at the mouse and rat A_3_AR. Currently, there is an increasing use of SBDD techniques to identify new chemotypes as hA_3_AR ligands.

## 2. Structural Probing of the A_3_ Adenosine Receptor

### 2.1. Site-Directed Mutagenesis

**Figure 3:**
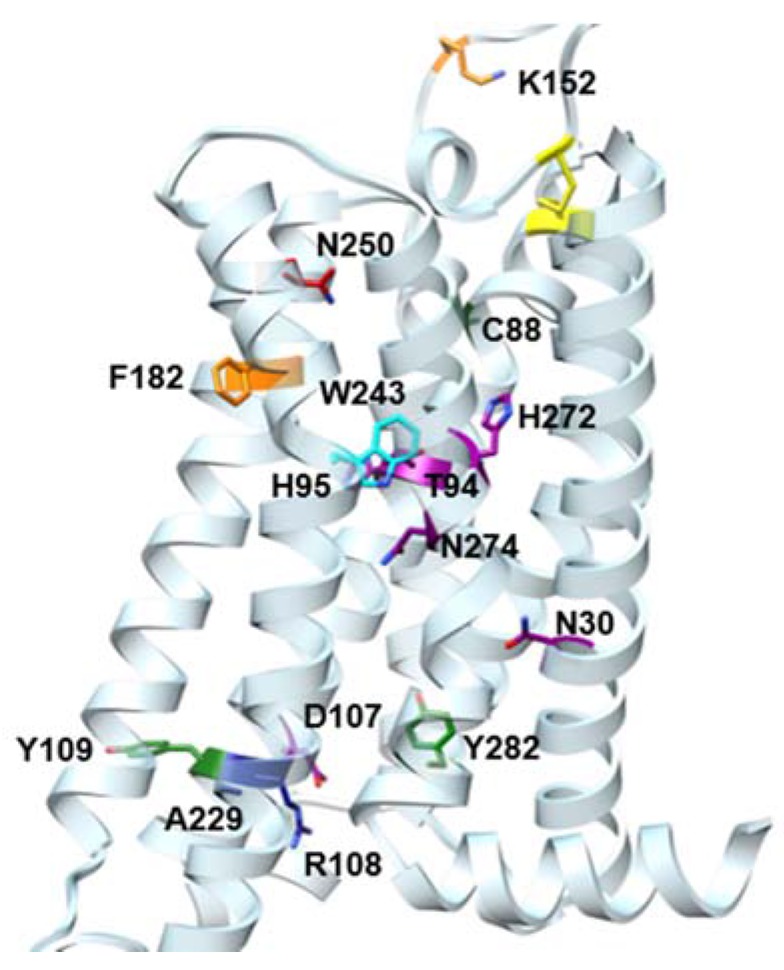
Summary of available hA_3_AR mutagenesis studies [14,15,16]. The color code is: green, mutation site affecting only agonist binding; orange, mutation site affecting only antagonist binding; purple, mutation site affecting both agonist and antagonist binding; blue, mutation site producing constitutively active mutants; red, Asn250 (6.55); cyan, Trp243 (6.48). The conserved TM3-EL2 disulfide bridge is also highlighted (yellow sticks). The residue Ballesteros–Weinstein notation [32] is: Asn30 (1.50), Cys88 (3.30), Thr94 (3.36), His95 (3.37), Asp107 (3.49), Arg108 (3.50), Tyr109 (3.51), Lys152 (EL2), Phe168 (EL2), Phe182 (5.43), Ala229 (6.34), Trp243 (6.48), Asn250 (6.55), His272 (7.43), Asn274 (7.45), and Tyr282 (7.53). TM, transmembrane α-helix.

Over the past 15 years, extensive SDM studies have been carried out at the hA_3_AR that led to the identification of residues playing a role in agonist and antagonist binding as well as in receptor activation. Collected mutational data are summarized in Table 1, while a graphical depiction, with a color code reflecting the effects of each mutation, is given in Figure 3.

The first hA_3_AR SDM study identified amino acid residues involved in the recognition of five agonists (as shown in Figure 1, adenosine derivatives 2-Cl-adenosine, **2**, and **9**; the North (*N*)-methanocarba derivative of **9** (MRS1898); and 5’-*N*-methyl 1,3-dibutylxanthine 7-β-d-ribofuronamide (DBXRM)) and four antagonists (*N*-[9-chloro-2-(2-furanyl)[1,2,4]triazolo[1,5-*c*]quinazolin-5-amine (CGS15943) and its *N*^5^-benzoyl derivative MRS1220; *N*-(2-aminoethyl)-2-[4-(2,3,6,7-tetrahydro-2,6-dioxo-1,3-dipropyl-1*H*-purin-8-yl)-phenoxy]-acetamide (XAC); and 4-methoxy-*N*-[2-(2-pyridinyl)quinazolin-4-yl]benzamide (VUF8504)) and dissected the role of two residues in TM6, namely Asn250 (6.55, using standard notation for numbering GPCR amino acid residues [32]) and Trp243 (6.48), that are conserved among all four ARs subtypes and in a variety of other GPCRs [16]. Asn250 (6.55) played an essential role for ligand recognition, as the Asn250Ala mutant receptor lost the ability to bind all the tested agonists and antagonists. Trp243 (6.48), which was proposed to act as a toggle switch in various other GPCRs as well as the A_3_AR [33], was critical for receptor activation and the accommodation of antagonists (but not agonists) in the orthosteric binding site (the site for the native ligand) through π-π stacking interactions. Compatibly with this hypothesis, the Trp243Ala mutation affected to a greater extent the binding of antagonists bearing phenyl substituents (MRS1220 and VUF8504) with no clear distinction among different chemotypes. Two histidine residues in TM3 and TM7, His95 (3.37) and His272 (7.43), were required for both antagonist and agonist binding with the effect of their mutations being related to the chemotype of the tested ligands: indeed, the His95Ala mutation affected the binding of 2-Cl-adenosine and (*N*)-methanocarba derivative MRS1898, whereas His272Glu prevented the binding of xanthine riboside DBXRM (agonist) and 8-phenylxanthine derivative XAC (antagonist). Interestingly, the binding of MRS1220, a triazolo-quinazolin-acetamide derivative bearing both a chloro and an *N*^5^-benzoyl substituent was reduced by 600-fold by the His95Ala mutation while being abolished by the His272Glu mutation. Another site affecting only antagonist binding with no distinction between chemotypes was identified in the Lys152Ala (EL2) mutation.

Another SDM study identified residues participating in the response of the hA_3_AR to pyridinylisoquinoline (**24**, **25**) and imidazoquinolinamine (**26**) derivatives (Figure 2) that act as PAMs [14]. Recognition of these PAM classes was associated with a different set of residues, although with some overlap, with respect to those required for orthosteric ligands. A_3_AR PAMs slow the dissociation rate of agonist radioligands, which is the most reliable method to characterize and compare new PAMs [14]. Among the tested mutation sites, Phe182 (5.43) and Asn274 (7.45) were required for the modulation mediated by all PAMs. On the other hand, the different effect produced by the Asn30Ala (1.50), Asp58Asn (2.50), and Asp107Asn (3.49) mutations, all located in the intracellular portion of the TM bundle and more likely to affect ligand binding through indirect effects, suggested that the two PAM classes might adopt different binding modes at the hA_3_AR.

SDM was also exploited to identify amino acid residues in the conserved motif of the tripeptide sequence DRY (TM3) and in TM6 whose mutation produces hA_3_AR constitutively active mutants (CAMs) [15]. The Ala229Glu (6.34), Arg108Ala (3.50), and Arg108Lys (3.50) receptor mutants displayed enhanced agonist-independent activity, while binding agonists with an affinity equal to or greater than the wild-type receptor. These mutations presumably maintain the hA_3_AR in an active conformation able to couple with a G protein. Other tested mutations, such as Cys88Phe (3.30), Tyr109Phe (3.51), and Tyr282Phe (5.43), greatly decreased the potency of the agonist.

### 2.2. Homology Modeling

As mentioned in the introduction, an A_3_AR X-ray structure is not yet available. Until the recent determination of the structure of a closer homologue [10], A_3_AR homology models were mostly derived by using the 2.8 Å resolution structure of bovine rhodopsin as a template (Protein Data Bank (PDB) code: 1F88) [34]. The disclosure of the first X-ray structure of an AR family member [10], namely the hA_2A_AR, provided a more accurate template and contributed to an overall improved structural representation of all the ARs. Though characterized by a higher sequence identity with the hA_3_AR (~40%) than with the previously used templates, the hA_2A_AR X-ray structures present unique features that might impact the shape, as well as the physicochemical properties of the orthosteric binding site and of the homology models derived from it. Indeed, the hA_2A_AR features EL2 with three disulfide bridges (through Cys71–Cys159 and Cys74–Cys146 linking EL1 to EL2, and Cys259–Cys262 within EL3), in addition to the disulfide linkage between EL2 and TM3 that is conserved among most of the class A GPCR members [35,36]. The additional disulfide bridges contribute to form a rigid lid that exposes the hA_2A_AR orthosteric binding site to the solvent. The hA_3_AR, on the contrary, has only one cysteine residue in EL2 (Cys166), which is expected to form the conserved disulfide bridge to Cys83 (3.25) in TM3 (yellow sticks in Figure 3). It is therefore conceivable that the A_3_AR exhibits a higher plasticity in the EL region with respect to the hA_2A_AR, and that A_3_AR homology models might need a careful validation and refinement in order to ensure they effectively take into account this feature.

**Figure 4:**
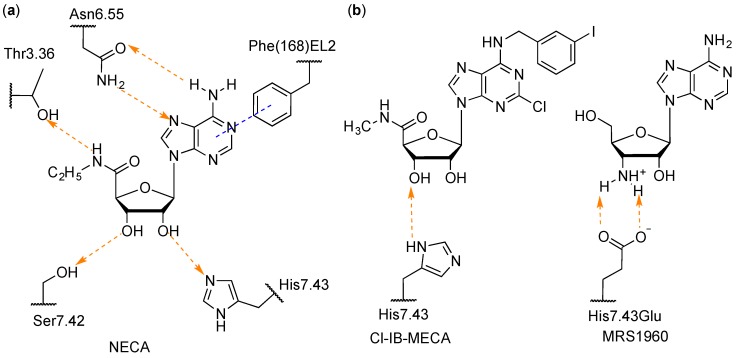
(**a**) Contacts of NECA **2** when docked in the hA_3_AR (using Ballesteros–Weinstein notation [32]). The residue numbers are Thr94 (3.36), Asn250 (6.55), His272 (7.43) and Ser271 (7.42); (**b**) Schematic depiction of the neoceptor approach at the A_3_AR: comparison of the binding of Cl-IB-MECA **9** at the wild-type hA_3_AR (left) with the binding of neoligand MRS1960 **3** at the His7.43Glu mutant A_3_AR (right) [24]. Orange arrows denote H-bonds, while blue lines π-π stacking interactions.

To date, the X-ray structures of the hA_2A_AR in complex with four different agonists have been solved (PDB codes: 2YDV, 2YDO, 3QAK and 4UG2) [10,35,36,37]. The first agonist-bound A_2A_AR structure reported was with UK432097 (3QAK) [35], which is an adenosine derivative (6-(2,2-diphenylethylamino)-9-((2*R*,3*R*,4*S*,5*S*)-5-(ethylcarbamoyl)-3,4-dihydroxytetrahydrofuran-2-yl)-*N*-(2-(3-(1-(pyridin-2-yl)piperidin-4-yl)ureido)ethyl)-9*H*-purine-2-carboxamide), with a NECA (**2**)-like ribose modification, that contains large, stabilizing *N*^6^ and C2 substituents. The UK432097 complex displayed greatly increased thermal stability (melting temperature (T_m_) elevated by 7 °C compared to the complex with CGS21680, 2-[*p*-(2-carboxyethyl)-phenyl-ethylamino]-5′-*N*-ethylcar-boxamidoadenosine). This extra stabilization obviated the need for receptor stabilization through a mutagenesis approach known as Stabilized Receptor (StaR^©^), as was used for the complexes with adenosine (**1**, 2YDO) and 5′-*N*-ethylcarboxamidoadenosine (NECA) (**2**, 2YDV) [36]. The interaction pattern that anchors potent nonselective AR agonist NECA (**2**) in the hA_2A_AR co-crystallized structure shows characteristic contact with residues, which, in many cases, is conserved in other ARs. However, it has to be pointed out that two key interactions that coordinate NECA in the A_2A_AR [36,37], i.e., the H-bond between His (6.52) and the 5′-carbonyl and the one linking Glu (EL2) with the exocyclic amine, are absent as these two residues occur as Ser (6.52) and Val (EL2), respectively, in the A_3_AR (Figure 4a). It is therefore conceivable to expect that those two residues might play a role in determining the ligand selectivity profile based on the adenosine scaffold. Nonetheless, the predicted overall interaction pattern common to various nucleoside agonists at the A_3_AR is consistent with the placement of adenosine derivatives during a two-decade-long progression of knowledge of the AR binding site, based on empirical probing ligand SAR, molecular modeling, and mutagenesis analysis.

An early SAR study of the rat A_3_AR included a molecular model that was based on bacteriorhodopsin (PDB code: 1BRD), which correctly predicted the ribose ring to be deeply embedded in the binding site and in proximity to a conserved His in TM7 [38]. The existence of a sub-pocket surrounding the ribose moiety was suggested as indicative of a receptor activation switch to explain the conversion of a series of adenosine (riboside, 4′-CH_2_OH) derivatives with C2-(ar)-alkynyl chains, which were A_3_AR partial agonists, to full A_3_AR agonists by substitution or modification of a 5′-alkyluronamide. Thus, the hydrophilic and sp^3^ hybridized ribose moiety is required for activation of the A_3_AR by nucleosides and as such may be considered the message moiety for receptor activation, while the hydrophobic sp^2^ hybridized nucleobase constitutes the address moiety that determines receptor selectivity. The comparison between agonist- and antagonist-bound A_2A_AR structures shows that the ribose moiety displaces water molecules, which have been computationally predicted to be “unhappy” [39], from a deep sub-pocket surrounding the antagonist structure. The placement of the ribose moiety as predicted by docking at the A_3_AR homology models suggests that a similar phenomenon might occur in the A_3_AR as well. Nucleoside analogues that have portions of the ribose moiety truncated have progressively reduced A_3_AR efficacy, i.e., they are unable to accommodate, or induce, the receptor conformational change associated with full activation. For example, **16** displayed reduced efficacy as an antagonist in a functional assay of [^35^S]GTP-γ-S binding, however it retained agonist properties in adenylate cyclase inhibition [28]. Various isolated nucleobase analogues lacking the ribose moiety are known to bind with similar AR selectivity as the corresponding riboside but entirely lack the ability to activate the receptor [40]. Thus, the distinction of address and message portions of a GPCR ligand is as applicable to the small molecule adenosine, as to peptide hormones [41].

Consistent with the division of roles of the ribose and adenine moieties, certain ribose modifications consistently reduced the A_3_AR efficacy, such as removal of the 5′-amide NH in NECA-like agonists, either by *N*-alkylation to form a tertiary amide or by conversion of the amide to an ester, or by truncation of the amide entirely [7]. The NECA (**2**)-like 5′-alkylamide in agonists enhances A_3_AR affinity and enhances efficacy, and an *N*-methylamide is favored over *N*-ethyl- and *N*-cyclopropylamides. Although many ribose modifications reduce A_3_AR affinity, 4′-thionucleoside and (*N*)-methanocarba (bicyclo[3.1.0]hexane) modifications of ribose (e.g., **12**–**16**) are well tolerated at the A_3_AR [7]. The 4′-truncation of adenine nucleosides, both in the ribose and (*N*)-methanocarba series, reduces the efficacy of A_3_AR activation; thus, such truncated nucleosides such as **16** tend to be antagonists or low-efficacy partial agonists at the A_3_AR. Also, flexibility of the 5′-amide or CH_2_OH appears to be a requirement for A_3_AR activation, as steric constraint of the 5′-amide in the form of spirolactam **4** produced an antagonist that was a potent and selective antagonist the A_3_AR [6,21]. The A_2A_AR undergoes conformational tightening, driven by H-bonding, around the ribose moiety in the agonist-bound, active-like state [35]. Isomeric pairs of apioadenosine derivatives were µM in binding to the A_3_AR [22]. A comparison of the stereochemistry required for partial agonism at the receptor (an α-d-apio-l-furanoadenosine derivative, **5**), compared to antagonism (an α-d-apio-d-furanoadenosine derivative, **6**), correlated with the H-bonding ability of the ligand with Thr94 (3.36), as determined in docking to an A_3_AR homology model.

**Figure 5:**
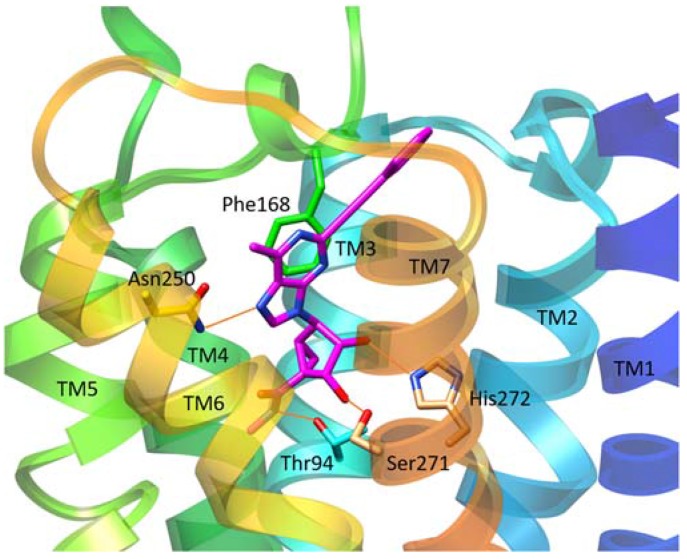
Docking pose of 6-Me analogue MRS5919 (**13**, magenta) at the hA_3_AR (hybrid homology model, A_2A_AR-β_2_ template) [23]. H-bond interactions are represented as orange solid lines. The residue Ballesteros–Weinstein notation [18] is: Thr94 (3.36), Phe168 (EL2), Asn250 (6.55), His272 (7.43), Ser271 (7.42) and His272 (7.43).

The ability to modify the nucleobase and usually still retain A_3_AR efficacy, either as partial or full agonist as long as an intact ribose is present, was demonstrated upon removal of the exocyclic amine or its replacement with an alkane or alkene, which is incapable of stabilizing the bidentate H-bond of N7 and *N*^6^H with Asn250 (6.55) [23]. The full A_3_AR agonist MRS5919 (**13**) (C6-CH_3_, Figure 5) docked in the receptor in the same orientation as the corresponding C6-NHCH_3_ analogue **14**. Thus, the lack of an otherwise conserved H-bond to the adenine moiety had no effect on the efficacy, although the binding affinity was reduced by 7-fold. Note that there is no H-bond present with N1, consistent with the ability of 1-deaza nucleosides to bind to the A_3_AR with minimal affinity loss.

The earliest modeling studies of the hA_3_AR were based on homology with the high-resolution structure of bovine rhodopsin [14,16,24,33]. The model featured orthosteric agonist binding with the hydrophobic adenine moiety and the substituted *N*^6^-benzyl group of **9** and its congeners directed to the EL region at the opening of the binding site and the ribose moiety in a deeper, hydrophilic region of the TM binding domains. Smaller *N*^6^ groups, such as methyl, are tolerated better at the hA_3_AR than at the mouse or rat A_3_AR, while the effects *N*^6^-benzyl groups tend to be generally of high affinity across species. In support of that ligand placement, a neoceptor approach was utilized (see following section) [24]. Rhodopsin-based homology models [42,43] were also able to identify an H-bonding network surrounding the ribose moiety of adenosine and leading toward the cytosolic side of the A_3_AR. The model was used to explain the enhanced affinity of 2-cyano-adenosine derivatives and the interdependence between the hA_3_AR regions accommodating the *N*^6^ and C2 groups, suggesting that a favorable orientation of the C2 group in the binding site was driven by additional hydrophobic interactions established by the phenyl ring of the *N*^6^ substituent [42].

### 2.3. Receptor Reengineering

The accuracy of the A_3_AR homology models, especially before the determination of the hA_2A_AR X-ray structures, has been subject to uncertainties and limitations due to the low sequence identity of the available templates [43]. Moreover, as noted in the previous section, A_3_AR homology models derived using the close homologue hA_2A_AR as template still entail potential limitations in the description of receptor plasticity in the EL region. The neoceptor approach is a strategy that has been devised to verify the accuracy of homology models, and in particular of rhodopsin-based models, while envisioning a potential application for gene therapy [44]. This approach made complementary structural changes on the ligand and the receptor that were predicted to be in proximity when the agonist was bound. The finding of enhanced binding affinity of a strategically modified nucleoside in the mutated receptor validated its proposed position in the binding site [24]. In the first application of this approach, the ligand contained an unnatural amino group on the ribose moiety and the hA_3_AR receptor contained an unnatural carboxylic group, in the form of a Glu residue. Specifically, 3′-amino-3′-deoxyadenosine MRS1960 (**3**) bound to the His272Glu (7.43) mutant A_3_AR with a 7-fold affinity enhancement (Ki 75 nM), which was consistent with the spatial proximity of the paired ionizable groups, i.e., the now positively charged ribose moiety and the extra negatively charged receptor (Figure 4b) [24]. In the native A_3_AR, a His (bearing a partial positive change) present in vicinity of the adenosine 3′ substituent would disfavor an amino group at that position. The A_3_AR homology model based on an inactive GPCR structure (rhodopsin), still provided useful insights into agonist recognition. A larger than expected gap between His272 and the 3′-OH of agonists (4.21 Å) was speculated to be the site of an interposed water molecule [30]. Retrospectively, it is more likely that the conformational tightening of the receptor residues around the ribose moiety in the agonist-bound structure [35] would explain this gap in the previous model.

Subsequent studies introduced additional modified ligands that demonstrated the same spatial proximity in carboxy-mutated A_3_ARs [25]. Although the initial ligand affinity enhancement in a neoceptor was based on an electrostatic attraction with an amino-nucleoside **3**, introducing new H-bonding groups was also found to be suitable [44]. Extended 3′-acetamide **7** bound to the His272Glu (7.43) neoceptor with a 700 nM Ki value (>20-fold enhancement compared to the wild-type A_3_AR). The 3′-Urea derivative MRS3481 **8**, which is capable of forming multiple H bonds, bound to the His272Glu (7.43) neoceptor with a Ki value of 220 nM (>100-fold enhancement compared to the wild-type A_3_AR). Thus, an enhancement of affinity of a strategically-modified ligand at a neoceptor need not be based on an electrostatic interaction. As a negative control experiment, introduction of a Glu residue at a more distant location of the A_3_AR, in TM3, produced no enhancement for **8**. Compound **8** also induced a cardioprotective response typical of the A_3_AR in cardiomyocytes that were transfected with the His272Glu (7.43) neoceptor. Thus, the urea-modified nucleoside ligand retained orthogonal agonist properties in a mutant A_3_AR, but not the wild-type A_3_AR.

## 3. Molecular Modeling of the A_3_ Adenosine Receptor

### 3.1. Docking

Docking has been extensively used to provide binding hypotheses of nucleoside and nonnucleoside ligands at A_3_AR homology models with the aim of guiding subsequent SAR studies. However, molecular docking has also been applied in a less canonical way to provide useful insight into agonist-induced receptor activation.

In an earlier study [33], two rhodopsin-based homology models, namely a “pure” model and one in which a putative Meta I state was induced by altering the Trp243 (6.48) side chain conformation, were used to compare the local conformational changes induced by the binding of the agonist Cl-IB-MECA (**8**) and the antagonists PBS-11 (**22**) and MRE3008-F20 (**23**) and to propose a receptor activation mechanism. The analysis suggested that the activation of the A_3_AR required the destabilization of the H-bonding network involving Trp243 (6.48) and His272 (7.43) and the one anchoring the ribose ring to Thr94 (3.36), Ser271 (7.42) and His272 (7.43), along with a rotamer switch of Trp243 (6.48) and the inward movement of Phe182 (5.43). In a subsequent study [45], by using a homology model based on the hA_2A_AR, the docking results further suggested a shift of the Trp243 (6.48) from an inactive “vertical” conformation to an active “horizontal” one upon agonist binding. As we will discuss in the following section, this conformational switch of Trp243 (6.48) has not yet been observed experimentally.

More recently, a docking analysis compared the binding modes of adenosine and NECA derivatives bearing C2-(aryl-alkynyl) groups acting as hA_3_AR partial and full agonists, respectively [18]. Compounds **10** and **11** were docked at a hA_3_AR homology model based on an antagonist-bound hA_2A_AR template and the resulting poses were subjected to short MD refinement. The analysis suggested that the C2-(aryl-alkynyl) group plays a role in the correct orientation of the 5′-substituent and that partial and full agonism might be linked to the agonist being able to adopt a *syn* or *anti* conformation of the glycosidic bond, respectively.

**Figure 6:**
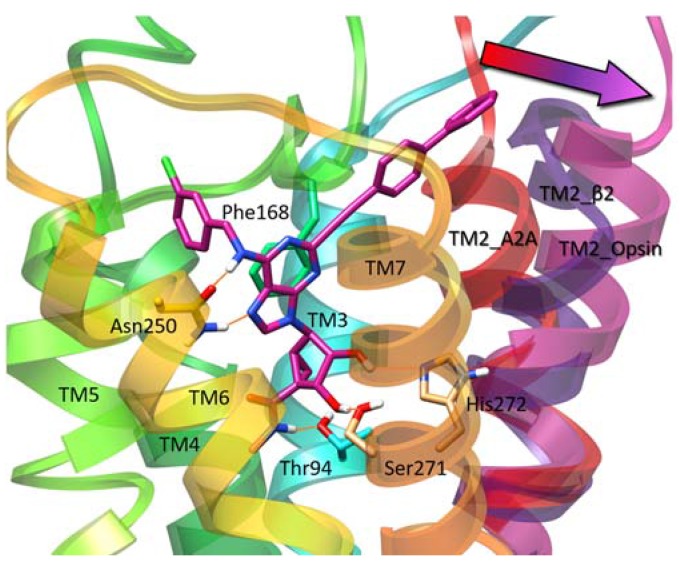
Docking pose of MRS5679 (**12**, violet sticks) at the hA_3_AR model based on a hybrid A_2A_AR-opsin template where TM2 (violet ribbon) is shifted outward from the binding site with respect to the model based on the hybrid A_2A_AR-β_2_ template (purple ribbon) and the one based entirely on the human A_2A_AR (red ribbon) [27]. H-bond interactions are represented as orange solid lines and TM1 is omitted to aid visualization. The residue Ballesteros–Weinstein notation [32] is: Thr94 (3.36), Phe168 (EL2), Asn250 (6.55), His272 (7.43), Ser271 (7.42) and His272 (7.43).

Another docking analysis explained the high A_3_AR selectivity of C2-extended (*N*)-methanocarba nucleoside analogues, such as biphenyl derivative **12**, with the hypothesis of receptor plasticity [26]. As emerged from the binding data, the A_3_AR, but not the A_1_AR or A_2A_AR, is highly permissive in a region extending toward the EL region that can accommodates a variety of C2-alkynyl and C2-(aryl-alkynyl) groups, as large as pyrenyl-alkynyl groups. A plausible structural explanation is that, to maintain the conserved H-bonding and π-π stacking interactions of the adenosine moiety, an outward movement of the upper portion of TM2 (with respect to its position based on an agonist-bound A_2A_AR template) is required. Upon overlaying the A_2A_AR structure with the helical orientations of the active states of the β_2_-adrenergic receptor and opsin, all the helices except for TM2 were closely approximated; however, TM2 was displaced outward. A hybrid model of the A_3_AR, with all helices except TM2 based on the A_2A_AR structure and TM2 based on opsin [26], was able to accommodate the C2-extended analogues without a steric clash. In the A_2A_AR, the presence of four disulfide bridges in the ELs would preclude such an outward movement of TM2, thus effectively preventing analogues like **8** from binding. The concept of receptor plasticity as well as the outward shift of TM2 has been also recently suggested to play a role in modulating signaling pathways [27]. A docking analysis of C2-functionalized (*N*)-methanocarba 5′-*N*-methyluronamides that were biased agonists favoring cell survival in comparison to other functional readouts, using hybrid A_3_AR models bearing different TM2 displacements, suggested that a progressive outward TM2 shift was required to accommodate the C2 substituents (Figure 6). Interestingly, the degree of signaling bias towards cell survival was dependent upon the extension of the C2 moiety (the longer the substituent, the greater the bias), and directly correlated with the proposed outward shift of TM2 within a 2–12 Å range [27]. It should be noted that docking of C2-extended A_3_AR nucleoside antagonists also required an outward TM2 movement [46]; thus, both agonist and antagonist-bound states require a hybrid A_3_AR model [26]. Notably, this hypothesis has been confirmed by the recent X-ray structure of the A_1_AR in complex with an irreversible antagonist, in which TM2 was displaced by about 5 Å with respect to the TM bundle [47].

**Figure 7:**
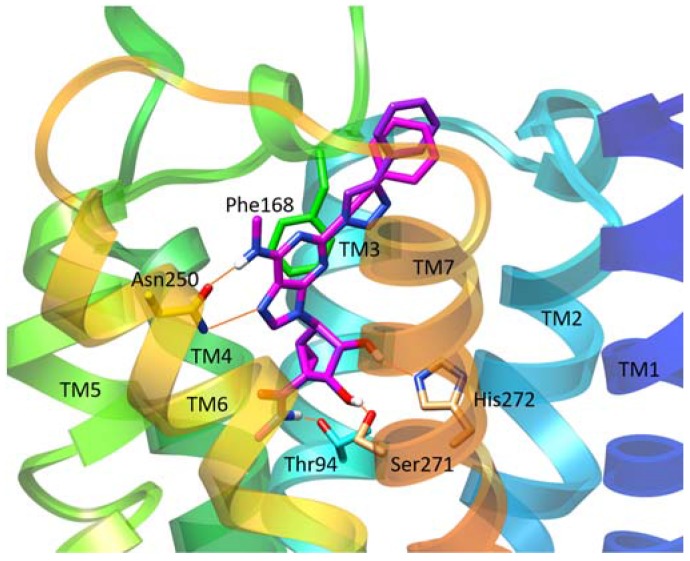
Comparison of the docking of a 2-(phenylethynyl) agonist MRS5644 (**14**, magenta) and the corresponding 2-(phenyl-triazolyl) agonist MRS7110 (**15**, purple) at the hybrid hA_3_AR model based on a A_2A_AR-β_2_ template [48]. The positions of the terminal phenyl rings overlap to a large degree. The residue Ballesteros–Weinstein notation is: Thr94 (3.36), Phe168 (EL2), Asn250 (6.55), His272 (7.43), Ser271 (7.42) and His272 (7.43).

A docking analysis also served the purpose to search for linkers to replace the alkyne group of the highly selective A_3_AR agonists such as MRS5644 (**14**), because of potential toxicity associated with certain arylalkynes. A variety of one- and two-carbon groups (methylene, alkene, etc.) and small cyclic linkers were compared, using docking in the hybrid A_3_AR homology model [48]. The objective was to retain the preferred geometry of the terminal alkyne with respect to the adenine moiety when bound in the A_3_AR. The triazole linker in compound MRS7110 (**15**), which was readily synthesized by a click reaction, was found to have a predicted overlap with the alkyne when docked in the receptor binding site (Figure 7). Compound **15** was found to have similar affinity (K_i_ at hA_3_AR = 0.96 nM) and A_3_AR selectivity with respect to the parent alkyne **14** (K_i_ at hA_3_AR = 0.85 nM).

### 3.2. Molecular Dynamics (MD)

With respect to other GPCRs, only very few studies describing MD analysis of A_3_AR have been reported in literature. The first MD simulation of an agonist-hA_3_AR complex embedded in a phospholipid bilayer [19] examined the conformational changes of conserved Trp243 (6.48). The study suggested that the agonist Cl-IB-MECA (**9**) but not the antagonist PBS-10 (**21**) stabilized the *trans* conformation of the Trp243 (6.48) residue, and that the conformational switch was presumably driven by a gain in the electrostatic interaction energy. Although from SDM experiments it is clear that this residue plays a role in receptor activation [33], and MD analyses conducted at both the hA_3_AR [19] and the hA_2A_AR [49] suggested that a conformational switch might be involved in the process, no side chain rotation has been detected in the active X-ray structure that was recently solved [50]. This might imply that either this residue has a different role in receptor activation or that the conformational switch might result in a transient species difficult to capture experimentally.

**Figure 8:**
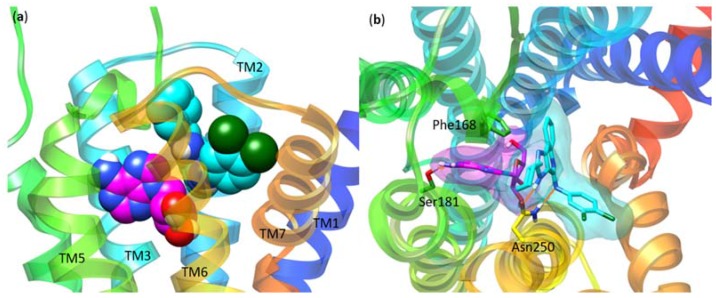
Representation of the ternary complex between hA_3_AR, the endogenous agonist adenosine (**1**, magenta) and the positive allosteric modulator LUF6000 (**25**, cyan) as obtained by supervised molecular dynamics (MD) simulation [20]: side view (**a**) and top view (**b**). The residue Ballesteros–Weinstein notation [32] is: Phe168 (EL2), Asn250 (6.55), and Ser181 (5.42).

More recently, Supervised MD (SuMD) has been used to model the approach of the selective imidazoquinolinamine PAM LUF6000 (**25**) to its putative allosteric binding site at the hA_3_AR [20]. In the SuMD approach [51], a series of short unbiased MD simulations is performed, in which a tabu-like algorithm monitors the distance between the barycenters of the ligand and the binding site. Depending on the supervision outcome, i.e., if the ligand-binding site distance is shortening or not, the simulation is restarted either from the last coordinate set or from the original one, respectively. The supervision is repeated until the ligand-binding site distance is less than 5 Å and enables to visualize possible ligand–target approaching paths in a nanosecond simulation time scale. The system modeled in the SuMD simulation [20] considered LUF6000 approaching the hA_3_AR in complex with the endogenous agonist adenosine (**1**). Prior reaching the orthosteric site, the PAM interacted with a meta-binding site located at the interface between EL2 and the upper region of TM5 and TM6. The recruitment of Phe168 (EL2) to establish a π-π stacking interaction with LUF6000 pushed the agonist deeper in the orthosteric binding site, thus accommodating the PAM. In the final ternary complex (Figure 8), the agonist recovered the π-π stacking interaction with Phe168 (EL2), and LUF6000 established a bidentate H-bond interaction with Asn250 (6.55) and several hydrophobic contacts with residues in the upper region of the orthosteric binding site, while directly interacting with adenosine acting as “pocket cap”.

## 4. Library Screening to Discover Novel Ligands of the A_3_ Adenosine Receptor

**Figure 9:**
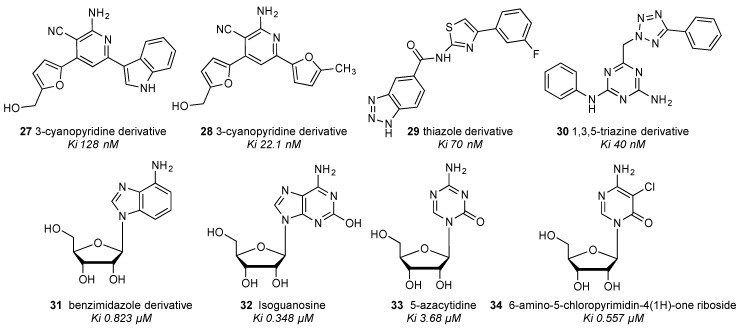
Structures of novel nonnucleosides (**27**–**30**) and nucleosides (**31**–**34**) found to bind to the hA_3_AR (Ki values given) as a result of structure-based virtual screening (VS) [52,53,54].

Although to date VS campaigns focused on the identification of novel A_3_AR binders have not been conducted yet, diverse chemical library screening at the orthosteric binding sites of A_2A_AR X-ray structures and of homology models of closely related AR subtypes has resulted in the discovery of novel A_3_AR ligands (Figure 9).

Screening a library of 6.7 million commercially available compounds at the agonist-bound A_2A_AR X-ray structure identified compounds **27** and **28** as novel albeit non-selective A_3_AR antagonists [52]. Although the template used was based on an active-like state of the A_2A_AR (at least in the ligand vicinity), no nonnucleoside AR agonists were discovered by that docking approach.

Screening a library of 2.2 million lead-like compounds at four different variants of an A_1_AR homology model based on an A_2A_AR antagonist-bound structure identified **29** and **30** as novel A_3_AR-selective antagonists [53]. In addition, novel antagonists of mixed AR selectivity were discovered.

An alternative strategy to discover new AR agonists by VS conceptually separated the ribose and nucleobase moieties and screened a virtual library of ribosides at the agonist-bound A_2A_AR structure [54]. Thus, commercially-available nucleobase alternatives were screened in the orthosteric binding site by constraining the ribose conformation as observed in the X-ray complex. The nucleobases were selected by filtering out the ZINC library (http://zinc.docking.org) for nucleobase-sized heterocycles with an available nitrogen atom for condensing chemically to a ribose moiety. The resulting focused library contained ~7000 small molecules, i.e., nucleobases, thus greatly increasing the possible diversity in comparison to the limited number of preformed nucleosides available in the commercial library (~300). The selected hits were synthesized and subjected to binding assays at hARs. Of the 13 ribosides that were screened for binding activity at A_1_AR, A_2A_AR and A_3_AR, 9 compounds displayed significant activity at one or more of the ARs (69% success rate), and several of these represented atypical agonist scaffolds. This process led to the discovery of nucleosides **31**–**34** as A_3_AR ligands. Nucleoside **31** was A_3_AR-selective in binding (Ki 800 nM), while **32** and **34** were full agonists at both A_1_AR and A_3_AR. Nucleoside **33** was a weak partial agonist at A_3_AR, and it also bound to A_1_AR.

## 5. Receptor Dimerization

Family A rhodopsin-like GPCRs are known to form dimers, either homodimers or heterodimers, which display characteristic pharmacology due to allosteric interactions between the receptor protomers. However, to date, the pharmacological relevance of receptor dimers is still not well understood. A common approach to investigate the structure and function of receptor dimers is through the design of bivalent ligands, i.e., ligands consisting of two pharmacophoric units, each targeting a protomer, linked by a proper spacer [55]. While serving as pharmacological tools to unravel the significance of receptor dimers, bivalent ligands hold promise to represent safer drug candidates. The existence of an A_3_AR homodimer in a single cell was demonstrated by measuring the dissociation kinetics of a fluorescent agonist, and negative cooperativity was shown to occur through the dimer interface [56]. In early molecular modeling studies on a rhodopsin-based model, TMs 4 and 5 were predicted to form the A_3_AR homodimer interface [57]. Notably, those TMs were involved in an extensive interface between two receptor units in the hA_1_AR X-ray structure recently solved [47]. Based on the hypothesis that A_3_AR might participate in heterodimer formation, tethered bivalent A_1_AR and A_3_AR agonists were synthesized, and the conjugates displayed high affinity in binding at both receptors and were cardioprotective as expected for each type of agonist [58]. However, despite this evidence, to date, the structural validation of bivalent ligands targeting A_3_AR dimers is mostly unexplored.

## 6. Conclusions

**Figure 10:**
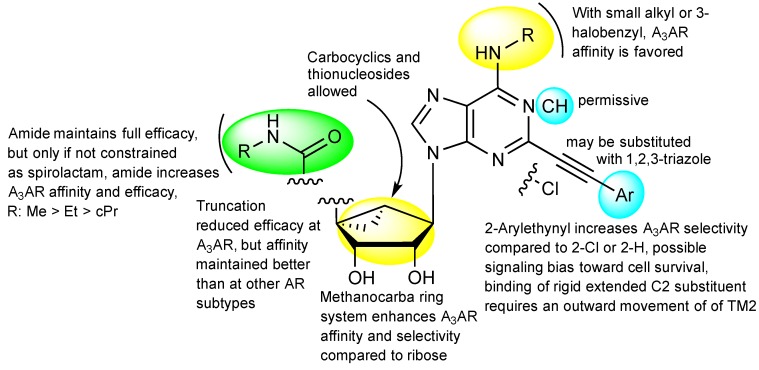
A general SAR summary of nucleosides at the hA_3_AR, including effects on affinity, efficacy, selectivity and receptor conformation.

Over the last two decades, the interplay between structure-based molecular modeling techniques, SDM, and empirical SAR of ligands has provided useful insights on the A_3_AR activation mechanism. The SAR of nucleosides binding at the A_3_AR has been probed in great detail, as summarized in Figure 10. This has led to the rational design of potent and selective A_3_AR agonists, mainly based on the nucleoside scaffold, and has laid the foundation for an improved understanding of more complicated mechanisms such as allosteric modulation and biased agonism. Conversion of agonists into partial agonists and antagonists was accomplished mainly by structurally altering the ribose “message” moiety of the nucleosides.

Notwithstanding, there are still challenges that need to be overcome, such as the improvement of the ability of homology models to reconstruct loop regions that are subjected to a higher structural variability with respect to the conserved TM architecture, and the design of VS strategies able to achieve the desired subtype selectivity. It will also be informative to probe the determinants of ligand residence time [59] on the A_3_AR as a rational approach to enhancing clinical efficacy and explore potential applications of bivalent ligands binding at receptor heterodimers.

Hopefully, the availability of three-dimensional A_3_AR structures in complexes with different ligand types will reveal interesting and unexpected structural details and resolve some hypotheses that have been raised so far. The design of novel A_3_AR agonists and other ligands, using structural insights and their translational development, promises to fulfill unmet medical needs [5,29,60].

## Figures and Tables

**Table 1:** Summary of Site-Directed Mutagenesis Analyses at the hA_3_AR.

Residue	Residue Number ^1^	Mutation	Effect on Binding of ^2^		Reference
			Agonist	Antagonist	
Asn30	1.50	Ala	Decreased	Decreased	[14]
Asn58	2.50	Asn	No changes	No Changes	[14]
Cys88	3.30	Phe	Decreased	No effect	[15]
Thr94	3.36	Ala	Decreased	Decreased	[14]
His95	3.37	Ala	Decreased	Decreased	[14]
Asp107	3.49	Asn	Minor changes	Decreased	[14]
Arg108	3.50	Arg,Lys,Asn, Glu,His	Minor changes	No effect	[15]
Tyr109	3.51	Phe	Decreased	No effect	[15]
Lys152	(EL2)	Ala	No effect	Decrease	[16]
Phe182	5.43	Ala	No effect	Decreased	[14]
Trp243	6.48	Ala,Phe	No effect ^3^	Decreased	[16]
Asn250	6.55	Ala	No binding	No binding	[16]
His272	7.43	Glu	Decreased	Decreased	[16,17]
Asn274	7.45	Ala	Decreased	Decreased	[14]
Tyr282	7.53	Phe	Decreased	No effect	[14]

^1^ [32]; ^2^ See text for chemotypes affected; ^3^ The ability of nucleosides to activate the receptor was progressively lost upon mutation to smaller residues. EL, extracellular loop.
